# Improvement of Liver Cell Therapy in Rats by Dietary Stearic Acid

**DOI:** 10.7508/ibj.2016.04.005

**Published:** 2016

**Authors:** Nasser Hashemi Goradel, Mohammad Ali Eghbal, Masoud Darabi, Leila Roshangar, Maryam Asadi, Nosratollah Zarghami, Mohammad Nouri

**Affiliations:** 1 Department of Medical Biotechnology, School of Advanced Medical Sciences, Tabriz University of Medical Sciences, Tabriz, Iran; 2 Department of Pharmacology and Toxicology, Faculty of Pharmacy, Tabriz University of Medical Sciences, Tabriz, Iran; 3 Liver and Gastrointestinal Disease Research Center, Tabriz University of Medical Sciences, Tabriz, Iran; 4 Department of Anatomy and Histology, Faculty of Medicine, Tabriz University of Medical Sciences, Tabriz, Iran

**Keywords:** Stearic acid, Liver failure, Transplantation

## Abstract

**Background:**

Stearic acid is known as a potent anti-inflammatory lipid. This fatty acid has profound and diverse effects on liver metabolism. The aim of this study was to investigate the effect of stearic acid on markers of hepatocyte transplantation in rats with acetaminophen (APAP)-induced liver damage.

**Methods:**

Wistar rats were randomly assigned to 10-day treatment. Stearic acid was administered to the rats with APAP-induced liver damage. The isolated liver cells were infused intraperitoneally into rats. Blood samples were obtained to evaluate the changes in the serum liver enzymes, including activities of aspartate aminotransferase (AST), alanine aminotransferase (ALT) and alkaline phosphatase (ALP) and the level of serum albumin. To assess the engraftment of infused hepatocytes, rats were euthanized, and the liver DNA was used for PCR using sex-determining region Y (*SRY*) primers.

**Results:**

The levels of AST, ALT and ALP in the serum of rats with APAP-induced liver injury were significantly increased and returned to the levels in control group by day six. The APAP-induced decrease in albumin was significantly improved in rats through cell therapy, when compared with that in the APAP-alone treated rats. *SRY* PCR analysis showed the presence of the transplanted cells in the liver of transplanted rats.

**Conclusion:**

Stearic acid-rich diet in combination with cell therapy accelerates the recovering of hepatic dysfunction in a rat model of liver injury.

## INTRODUCTION

Liver plays a major role in key processes of detoxification, homeostasis, glucose metabolism, protein synthesis, cholesterol metabolism, immune defense and bile secretion[1-3]. Hepatocytes are the main cells in liver and comprise 65% to 80% of the cellular population[4]. Metabolic dysfunction in these cells can cause hepatitis, cirrhosis and hepatocellular carcinoma[5].

Liver-related diseases place a heavy burden on socioeconomics and affect about 1.7% of the population of the world. Orthotropic liver transplantation, which is the gold standard interference for the end-stage liver disease[5,6], has disadvantages such as the limited availability of donors, risk of liver rejection and need for immunosuppression and sophisticated technology, as well as for long-term recovery[5-7]. These obstacles make the cell therapy a promising alternative to liver transplantation. The main advantage of this method is minimal invasiveness of the procedure[8,9].

The efficacy of current cell therapy for liver is not entirely satisfactory in terms of survival and function of the transplanted cells[10,11]. Initial steps of liver cell therapy are very important for the success of transplantation. Metabolic state of a host is the major determinant in the initial steps toward regenerating a functional liver. There are very few studies focusing on the effect of nutritional and metabolic status on the success rate of liver cell therapy.

Stearic acid is a common nutritional long-chain fatty acid and is known as a potent anti-inflammatory lipid[12]. On the contrary, other saturated fatty acids such as myristic acid and palmitic acid are positively associated with inflammation[13]. Stearic is the third most abundant fatty acid in human hepatocytes[14], and it is related to several liver functions, including cholesterol metabolism[15] and lipoprotein biogenesis[16]. Recent data have indicated that unlike oleic acid and linoleic acid, dietary stearic acid reduces adiposity[17] and inhibits cancer growth[18]. These effects have been attributed to a selective apoptosis impact of stearic acid on preadipocytes and cancer cells[17]. Dietary stearic acid increases serum oleic acid most possibly through the activation of hepatic enzymes[19].

Until now, there is no available data on the influence of stearic acid on liver cell therapy. Considering the profound impact of stearic acid on liver function, the present study investigated the effect of dietary stearic acid on the markers of hepatocyte transplantation in a rat model of liver damage induced by acetaminophen (APAP, N-acetyl-p-aminophenol).

## MATERIALS AND METHODS

### Animals and treatments

In total, males (n=5) and females (n=40) Wistar rats with an average weight of 250 g were used in this study. The rats were purchased from the Animal House of the Tabriz University of Medical Sciences, Tabriz, Iran. Animals were kept under a 12-h day and night rhythm and received a standard rodent diet with free access to water *ad libitum*. The male rats were used as hepatocyte donors and the female rats as recipients. The animals were handled and used according to the animal handling protocol, approved by a local ethics committee at Tabriz University of Medical Sciences, Tabriz, Iran.

Hepatotoxicity in female rats was induced by intraperitoneal administration of APAP (Sigma-Aldrich, St. Louis, USA) in a single dose of 1 g/kg. To potentiate APAP toxicity, phenobarbital was co-administrated in drinking water until the last day of the study (day 10)[20]. Animals were euthanized at day 10 after APAP administration. In stearic acid group, 180 g/kg stearic acid food pellet was fed from day 0 until the end of the experiment (day10). This level of dietary stearic acid and duration were chosen based on similar research on the metabolic effect of oil ingestion in rat models[21]. It has previously been reported that major cell engraftment occurs as early as 3-5 days of transplantation[20,22]. Indeed, Rodrigues et al.[20] have demonstrated a detectable hepatocyte three days after transplantation.

### Hepatocyte isolation and transplantation

Hepatocytes were isolated from male Wistar rats by collagenase perfusion method as described previously[23]. In brief, the rats were anaesthetized by inhalation of diethyl ether. The livers were perfused through portal vein with collagenase (Type V collagenase, Sigma, St. Louis, MO, USA) and digested *in situ* in Hanks’ buffer (137 mM NaCl, 5.4 mM KCl, 4.0 mM NaHCO_3_, 1.7 mM CaClz, 0.8 mM MgSO_4_, 0.5 mM KHZPO_4_, 0.3 mM NaZHPO_4_ and 10 mM HEPES, pH 7.6), dissociated hepatocytes were separated by sedimentation. Freshly, isolated hepatocytes were suspended (1×10^6^ cell/ml) in Krebs-Henseleit buffer containing 12.5 mM HEPES. Cell viability was assessed by the estimation of plasma membrane disruption as determined by Trypan blue uptake test[24].

After 24 h of APAP administration, 1×10^7^ hepatocytes were injected intraperitoneally[20]. Female rats were randomly divided into four groups of 10 each: sham, APAP, APAP+hepatocyte transplantation and APAP+hepatocyte transplantation+stearic acid groups.

### Biochemical analyses

Blood samples were collected at days 0 (before the injection of APAP), 1, 2, 3, 6 and 10 following hepatocyte transplantation. The collected samples were centrifuged at 1000 ×g for 5 minutes to separate the serum, followed by storage at -20ºC. The samples were analyzed for alanine aminotransferase (ALT), aspartate aminotransferase (AST), alkaline phosphatase (ALP) and albumin using a Biochemistry Auto Analyzer (Alpha-Classic At plus).

### Assessment of cell engraftment

To detect infused male cells in the liver of female recipient rats, liver DNA was extracted with Trizol (Invitrogen, USA) at day 10. PCR of sex-determining region Y (*SRY*) gene was performed using the following primers: Forward: 5’AAGCGCCCCATG AATGCATT 3’ and reverse: 5’CAGCTGCTTGCTGA TCTCTG3’. The amplified products were visualized on 1.5% agarose gels and stained with ethidium bromide[20].

### Statistical analysis

Data were expressed as mean±SD. The analysis of variance (ANOVA) and student *t*-test were used to compare variables among experimental groups. Values of *P*<0.05 were considered statistically significant.

## RESULTS

Immediately after the isolation, the applied method resulted in a high recovery rate of 95-98% for hepatocytes. *SRY* gene was detectable in all rats with cell therapy. However, PCR analysis showed no significant difference in the levels of *SRY* gene between cell therapy alone and cell therapy stearic-fed groups.

**Fig. 1:**
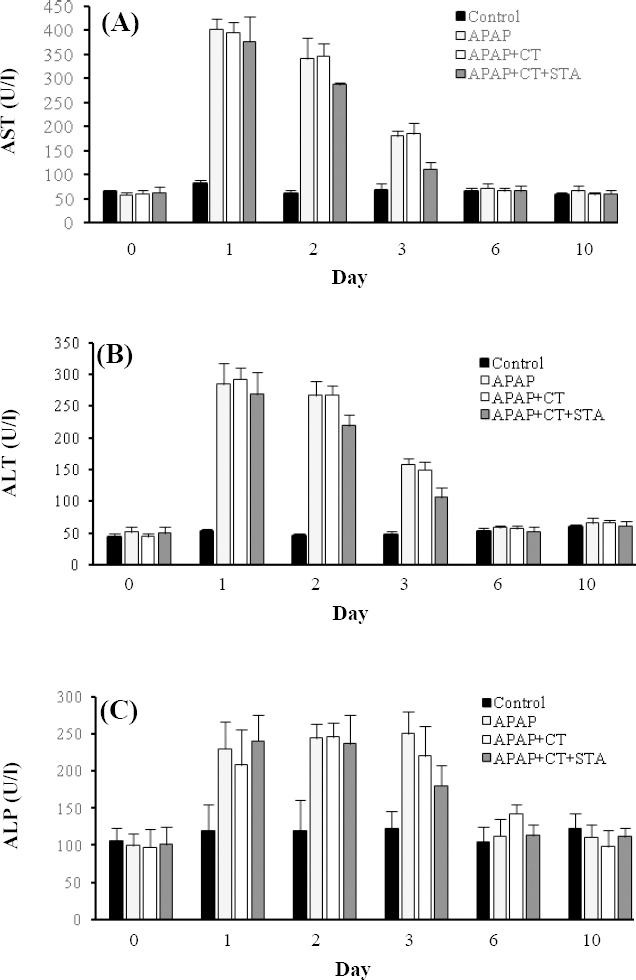
The effects of stearic acd (STA) combined with cell therapy (CT) on activities of serum (A) aspartate aminotransferase (AST), (B) alanine aminotransferase (ALT) and (C) alkaline phosphatase (ALP) in rats with acetaminophen (APAP)-induced liver damage. Data were mean±SD.

Serum levels of AST, ALT and ALP in rats with APAP-induced liver injury were significantly increased from day 1 and reduced over time (Fig. 1). In all experimental groups, serum activities of all liver enzymes returned to the levels in control group by day 6. Compared with cell therapy alone, rats in the cell therapy stearic-fed group showed more reduction in AST, ALT and ALP on day 3 (*P*<0.05).

**Fig. 2:**
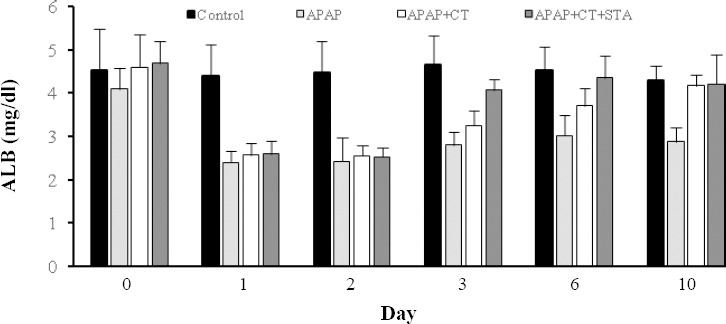
Effects of stearic acid (STA) combined with cell therapy (CT) on serum albumin (ALB) in rats with acetaminophen (APAP)-induced liver damage. Data were mean±SD.

Compared to the control group, APAP administration caused a significant decrease in albumin (~0.5-fold, *P*<0.001) and remained almost unchanged until the 10^th^ day (Fig. 2). The APAP-induced decrease in albumin was significantly improved in all rats with cell therapy when compared with that of the APAP-alone treated rats, restoring to control levels on day 10. However, on days 3 (*P*=0.002) and 6 (*P*=0.03), the cell therapy stearic-fed group showed a significantly higher albumin level than the cell therapy alone group.

## DISCUSSION

Hepatocyte transplantation is an alternative for orthotropic liver transplantation to cope with the lack of donors, immunological issues and operative and post-operative problems[5,6]. A single donor could be used for multiple recipients or multiple uses if necessary[9]. The present study focused on the effectiveness of hepatocyte transplantation on the liver function after liver damage by APAP and the role of stearic acid in hepatocyte transplantation.

Allogenic hepatocyte transplantation after liver damage accelerates liver regeneration. Studies have indicated the beneficial effects of saturated fatty acids, especially stearic acid, on hepatotoxicity[25,26]. These two avenues were investigated in the current study. A

rat hepatotoxicity model was used to show the positive effects of liver cell therapy, especially in stearic-fed rats, in comparison with control groups. Although the serum levels of ALT, AST and ALP were increased and albumin serum level decreased after APAP-induced liver damage, biochemical analysis showed the improvement of these markers in cell therapy groups, especially in stearic-fed rats. These findings suggest the key role of stearic acid in protection of liver against hepatotoxicity agents. This positive effect could be related to the potential anti-inflammatory role of stearic acid[27].

Pan and colleagues[28] have shown that stearic acid supplementation improves liver injury after bile duct ligation-induced liver injury in rats. Stearic acid can also attenuate induced liver inflammation by suppressing inflammatory cell recruitment/accumulation and/or NF-κB activity[28,29]. In addition, the increased level of NF-κB following liver injury elevates the expression of tumor necrosis factor-α, cyclooxygenase-2 and proinflammatory cytokines[29]. One study has demonstrated that the reduction in NF-κB after treatment with saturated fatty acid is accompanied by the increased amounts of IκBα, which is a stable form of the NF-κB inhibitor[30]. The assessment of pro-inflammatory transcription factors, enzymes and cytokines in experiments like these has been suggested to elucidate the mechanism of the positive effects of stearic acid on hepatocyte transplantation.

Unlike saturated fatty acids such as palmitic acid, stearic acid has a significant cholesterol-lowering effect[31]. The fast recovery of serum albumin to normal levels in stearic acid-fed rats could relate to this effect. Low cholesterol levels in the membrane after stearic acid treatment may improve homing of transplanted hepatocyte in the liver and facilitate the release of albumin into the blood.

Although previous research has revealed the beneficial effect of stearic acid on liver function, the present investigation is the first study to examine the impact of this saturated fatty acid on the hepatocyte therapy in a rat model. In this study, we measured several hepatic biomarkers in conjunction with the *SRY* assay to assess cell engraftment. Future studies may be conducted with longer-term follow-up and may focus on possible molecular mechanisms involved in favorable effect of stearic acid.

In conclusion, stearic acid-rich diet in combination with cell therapy accelerates the recovering of hepatic dysfunction in a rat model of liver injury. Data suggest that the beneficial effect of stearic acid is probably due to a favorable host metabolic status for cell retention.
